# The Plant Landscape of the “Conca d’Oro” of Palermo (NW Sicily, Italy) and Its Evolution

**DOI:** 10.3390/plants14060938

**Published:** 2025-03-17

**Authors:** Gianniantonio Domina, Giulio Barone, Enrico Bajona, Emilio Di Gristina, Giuseppe Venturella, Raimondo Pardi

**Affiliations:** 1 Department of Agriculture, Food and Forest Sciences, University of Palermo, Viale delle Scienze Bldg. 4, 90128 Palermo, Italy; gianniantonio.domina@unipa.it (G.D.); enrico.bajona@unipa.it (E.B.); emilio.digristina@unipa.it (E.D.G.); giuseppe.venturella@unpa.it (G.V.); raimondo.pardi@unipa.it (R.P.); 2 National Biodiversity Future Center (NBFC), University of Palermo, Piazza Marina, 61, 90133 Palermo, Italy

**Keywords:** urban biodiversity, ecosystem services, landscape history, spatial analysis, wetlands, plant conservation

## Abstract

The Conca d’Oro of Palermo, a plain in NW Sicily of significant historical and agricultural importance, has undergone significant landscape alterations due to agricultural strengthening and urbanization. This paper analyses the evolution of the plant landscape from early human settlements to the present by integrating historical records, cartographic analysis, and floristic surveys. Three key periods of change were identified: Roman-era deforestation for cereal cultivation, the expansion of irrigated agriculture under Arab rule, and the dominance of citrus monoculture in the 19th century. Post-World War II urban expansion led to the loss of agricultural land and natural habitats, particularly wetlands and coastal dunes. Spatial analysis revealed a drastic reduction in semi-natural areas, with agricultural land giving way to urban sprawl. Floristic studies showed the persistence of endemic plant species in fragmented natural habitats alongside the local extinction of wetlands and coastal vegetation. The Oreto River, a river with a basin that extends into the territories of the municipalities of Altofonte, Monreale, and Palermo, remains a critical biodiversity reservoir, and most other natural ecosystems have been degraded. This research provides insights into the long-term interactions between human activities and biodiversity and offers a foundation for sustainable conservation strategies in Mediterranean urban and peri-urban environments.

## 1. Introduction

Inhabited centers and their surrounding territory have undergone the greatest changes by man, with the construction of buildings, roads, soil movements, and crops that radically characterize the landscape. Landscapes evolve over long-term trajectories, with their origins rooted in the distant past. Examining land use and land cover changes over extended time periods provides a deeper understanding of the processes involved [1]. UNESCO first introduced the concept of the Historic Urban Landscape (HUL) in 2005 through the Vienna Memorandum [2], emphasizing the conservation of historical cultural heritage and urban planning in areas where cultural and natural values have accumulated over time. A defining characteristic of historic urban landscapes is layering, as cities evolve dynamically over time. Through this process, elements from different historical periods accumulate and overlap, shaping the present urban landscape [3,4]. Urban extension and the resulting changes in land cover have occurred throughout the world to accommodate the migration of populations from rural areas [5]. Cities have historically been, and continue to be, major consumers of natural resources, including domesticated and wild plants. Their use for food, raw materials, and fuel has had substantial impacts on landscapes, intensifying deforestation and driving the expansion of agricultural lands and pastures [6]. This transformation is reshaping ecosystems on a global scale, intensifying the impacts of climate change, and posing significant challenges to the long-term survival of biodiversity, with repercussions that are expected to extend well beyond urban areas [7]. Measuring land use change is essential for addressing global societal challenges, including food security, climate change, and biodiversity loss [8].

Plants remain of primary importance for biodiversity and human life for the various ecosystem services that they provide, from air purification to water regulation to the supply of food to recreational and psychological aspects [9]. Recently, there has been increasing recognition of urban green spaces as vital reservoirs of biodiversity and key providers of essential ecosystem services [10]. Urbanization drives intricate eco-evolutionary dynamics on a small spatial scale due to deep alterations in landscape features and environmental conditions [11]. The most altered habitat is the aquatic one. Water regulates the places in which human settlements are established. It is used for drinking, washing, hygienic functions, irrigation, energy sources, raising livestock, and as a route of communication, and for several centuries, it has provided the driving force for many artisanal and industrial activities. At the same time, marshy environments can take away space from agriculture, slow down transport, and favor disease development. Thus, aquatic environments are those most subject to alterations, and their plants are those most susceptible to local extinctions. The investigation of the natural plant species still growing in urban and peri-urban areas is to be associated with habitat fragmentation; by restricting gene flow—whether through barriers to seed dispersal or pollinator movement—it can numerically and genetically impoverish existing populations [12].

Most of the human population is concentrated in cities and their suburbs and spends most of its life there. What remains natural in these areas? Do humans still enjoy the natural richness of these areas, or do they live in a completely artificial environment? We studied the evolution of the plant landscape of the Palermo Plain, a metropolis in southern Italy, historically called “Conca d’Oro” for the richness of its crops, starting from historical sources up to today. The evolution of the urban factories and agricultural practices in the Palermo Plain are quite well known [13,14]. However, there is a lack of synthesis that addresses the effects that these dynamics have had on the plant landscape and the plant heritage of this area.

This research is structured in three sections: (1) a historical bibliographical review that summarizes the changes in the territory from the first human settlements to the 18th century; (2) a recognition of soil use based on the analysis of historical maps and satellite images that covers the period from the 18th century to the present day; and (3) a floristic study in herbarium and in the field aimed at investigating the floristic emergencies among the locally extinct taxa and the endemic ones still existing in the investigated area. The objective was to reconstruct what was present in terms of plant landscape and heritage since the time of human settlement, their historical evolution, and what remains today. Special attention was given to the aquatic environment, reconstructing the historically known wetlands and verifying how many of them are still present today. By providing detailed insights into the development processes of the city of Palermo and its surroundings, this research will contribute to the development of sustainable urban and peri-urban biodiversity conservation management practices in the Mediterranean region. Furthermore, the findings will be applicable to other Mediterranean coastal cities’ surroundings.

## 2. Materials and Methods

### 2.1. Study Area

**Figure 1:**
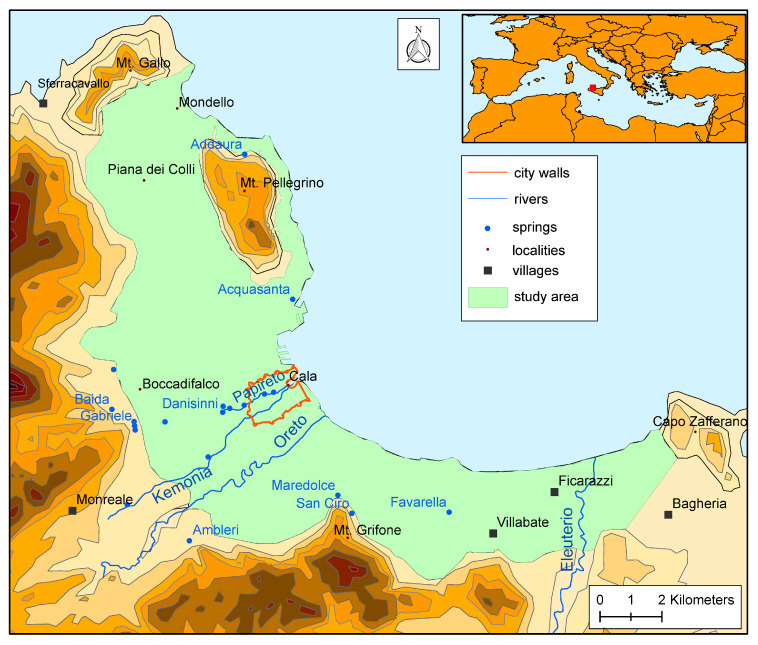
Area of investigation and its localization, with the main geographical elements reported in the text.

The Conca d’Oro is the plain on which the city of Palermo and some of the inhabited centers of its hinterland have developed. It is located in the northwestern part of Sicily (38°03′50″–38°12′14″ N and 13°17′13″–13°30′26″ E) and lies between the mountains of Palermo and the Tyrrhenian Sea. Monte Gallo constitutes the northwestern limit of the plain, and Monte Catalfano is the southeastern limit. The extension of the plain is about 100 km^2^. The plain has a slight slope, which ranges from 100 m a.s.l. at the foot of the mountains descending gently to the sea (Figure 1).

The Conca d’Oro falls mostly within the municipal territory of Palermo (≈650,000 inhabitants) and in the eastern part in the municipalities of Villabate (≈19,500), Ficarazzi (≈13,000), and to a small extent, Bagheria (≈53,000). The name “Aurea Concha” (Golden Basin) appeared for the first time in a 15th-century poem by Angelo Callimaco Siculo [15]. Similarly, this name is engraved on the base of a statue in the Palazzo Pretorio from around 1470, which represents “the Genius of Palermo”, the personification of the city [16].

The Palermo Plain was rich in water springs that emerged at the foot of the mountains and determined the formation of streams and wetlands. The main ones are the Oreto River (still present with an average annual flow of 0.86 m^3^/s), Eleuterio River (still present with an average annual flow of 0.5 m^3^/s), the Kemonia (buried in the 16th century), and the Papireto (buried in the 16th century) [17]. The spring-generated wetlands have been exploited over time, and marshy areas have been eliminated. The main ones are Acqua di Ambleri, Baida, Gabriele, San Ciro, Piana dei Colli, Acquasanta, Addaura, and Favare di Villabate (Figure 1). Over time, the terminal part of the river course has been straightened, and the riverbeds have been cemented. Another large wetland area was the brackish area of Mondello, which was reclaimed at the beginning of the 20th century.

### 2.2. Climate

According to Bazan and collaborators [18], the investigated area has a Mediterranean pluviseasonal oceanic bioclimate with a thermomediterranean thermotype and a dry-subhumid ombrotype in the bioclimatic classification [19,20]. The coldest month is January (average minimum = 7.8 °C, average maximum 15.7 °C), while the hottest is August (average minimum = 20.8 °C, average maximum = 29.9 °C). The annual average rainfall is 679.4 mm, with 78 rainy days [21,22].

### 2.3. Geomorphology and Pedology

The plain of Palermo belongs to the Sicilian segment of the Apennine–Maghrebian chain [23,24]. Rock outcrops include (a) Quaternary calcarenites (calcareous sands), which cover a large area of the Palermo Plain; (b) scree and debris, flows with accumulations of red earth of colluvial origin, mainly concentrated along the slopes of the relief; and (c) compact limestones rich in fossils. The Palermo Plain is made up of Pleistocene deposits (calcarenites and sandy clays) superimposed on the clay and marl soils of Numidic Flysch (Oligo-Miocene) [23,24].

Most of the territory has Red Mediterranean soils with reduced depth and fertility. Lithosols of very reduced depth and limited fertility are also abundant. The deepest and most fertile soils are alluvial and are found along the Oreto and Eleuterio Rivers in the depressions and southern and eastern areas of the Conca [25]. Man has made much of the land of the Conca d’Oro productive with irrigation, the addition of natural soil improvers, and, where necessary, as in some parts of the Piana dei Colli, by transporting new soil [26]. Over the last 10,000 years, the sea level along the coast of Palermo has remained almost stable, with maximum variations of 10 m more than the current level [27,28]. The most evident changes concern the burial and advancement of the coast at Cala due to the sediments brought by the Kemonia River [29]. Additionally, the coastline east of Cala advanced by approximately 200 m, as debris from buildings destroyed in the 1943 bombings was used to fill in sections of the sea [30].

### 2.4. Data Sources

Information on Palermo in the Arab–Norman period comes from the texts of Ibn Hawqal and Idrisi translated and commented on by Michele Amari [31,32]. Information about the Mediaeval period comes from Bresc [13], based on documents dating back to notarial funds of the 14th and 15th centuries.

### 2.5. Methods

This study follows three complementary approaches: a bibliographical review covering the period from the first human settlements to the 18th century, a spatial analysis based on the comparison of historical cartography and aerial photographs covering the period from the 18th century to the present day, and a floristic study covering the period from the 19th century to the present day.

#### 2.5.1. Spatial Analysis

Based on the available base maps, analyses were carried out with two different geographic focuses: one from 1754 to 2024 on an area of about 17 km^2^ centered on the city of Palermo, and the other from 1904 to 2024 on the entire Conca d’Oro.

For map georeferencing, at least 12 fixed elements for the sheet were identified from the Google aerial images of 2024 and used as Ground Control Points (GCPs). Historical buildings and road junctions were used as GCPs [33]. The maps, composed of multiple sheets, were georeferenced individually. The digitization of artificial areas is quite easy because their boundaries are readily distinguishable; areas of wetlands were clearly visible by their signs, but their boundaries on the map were unclear, making their digitization less accurate. The minimum vectorized unit was 25 linear m and 625 m^2^. Elements smaller than these dimensions were merged with adjacent ones. An attribute table was created for each shapefile in which essential characteristics, such as area, were calculated for each polygon, and all necessary land use information was added. The cartographic data were first converted into land use codes according to Corine Land Cover 3rd level [34] and then grouped into macro-categories for analysis.

The annual rate of change for each land use class was calculated using the formula suggested by Suleiman [35].

An assessment of Ecosystem Service Values over the years for the whole Conca d’Oro was conducted according to the methodology adopted by [36,37]. The updated Ecosystem Service Values of different land use classes referred to the Italian territory were retrieved from [38].

#### 2.5.2. Floristic Study

The study of the plant component lasted ten years. It was based on bibliographic studies and targeted field research. For the bibliographic study, the main local floras were consulted. The herbarium study was carried out at the *Herbarium Mediterraneum Panormitanum* (PAL), which is the primary data source for the area of Palermo [39]. The results were partially the subject of previous publications [40,41], whose results were integrated with field trips carried out monthly from 2022 to 2024. These trips were aimed at studying relict strips of semi-natural vegetation and searching for plant taxa of biogeographical interest. The prepared herbarium specimens were deposited at the herbarium of the Department of Agriculture, Food and Forest Sciences of the University of Palermo (SAF). The nomenclature of the taxa follows [42].

## 3. Results

### 3.1. Historical Review

The area of the city of Palermo has been inhabited since prehistoric times and has experienced at least 12 dominations throughout its history (Table 1).

Each of these civilizations contributed to the development of the city and the modification of its surroundings, not only with the construction of new buildings but, above all, with resource exploitation in the surrounding area. The first urban nucleus was established by local inhabitants in the 8th century BC in a flat area of about 50 ha delimited between the Kemonia and Papireto Rivers (Figure 1). A century later, the Carthaginians founded a trading base and built walls that were taken up and reinforced by the following dominations [16].

The plant landscape present at the time of the first human settlements was characterized by continuous plant cover. The different edaphic characteristics of the area led to different potential vegetation, i.e., lowland forest dominated by *Quercus pubescens* Willd s.l. in the deep and alluvial soils near the watercourses [45]; humid areas and marshes with rushes and Cyperaceae along the watercourses; *Q. ilex* L. forest in the rest of the plain on the deepest and most humified soils [46]; maquis with *Olea europaea* L. and *Pistacia lentiscus* L. in marginal, more xeric, and draining areas [47]; maquis with *Chamaerops humilis* L. along the coast; and dune vegetation in correspondence with the stretches of sandy coast.

The impact of human activities and related deforestation was limited to the immediate neighborhood of the settlement. The first major deforestations occurred during Roman domination, which invested huge amounts of capital to create large estates and wide properties dedicated to cereal cultivation [48]. During Roman rule, the city had more than 25,000 inhabitants [49]. Romans introduced the cultivation of the carob tree [50]. Grapes and olives had a more limited diffusion. Citrons, almonds, mulberries, pomegranates, and other fruits and horticultural crops had a narrow circulation and were cultivated for local consumption [51,52]. During the Byzantine domination, the population did not grow [49]. The territory continued to be characterized by large estates owned by the nobility and the church and cultivated with durum wheat [52].

During the Arab domination around the city of Palermo and inside the walls, there were natural springs from which streams flowed. These water courses were used for drinking, to irrigate vegetable gardens and orchards, and to operate mills. Around the city, there were small properties with vineyards and fruit gardens whose irrigation was guaranteed by canalized water from streams, water wells, or artificial underground water channels (Qanats). The Oreto River (Wadi Abbâs) was used to operate mills but not for irrigation or drinking. There were marshy lands along riverbeds; in the one along the Papireto stream, *Cyperus papyrus* L., which was used to make ropes, baskets, and sheets of paper, was grown [31]. Mondello was a marshy area used as a mooring [32,53]. Sugarcane (*Saccharum officinarum* L.) was probably brought to Sicily by the Arabs during their domination, but the little documentation on the subject suggests that it was a minor crop [54]. Records for “Persian cane” along the Papireto by Ibn Hawqal [31] could refer to sugarcane but also to giant cane (*Arundo donax* L.).

Toward the end of the 10th century, the city had 250,000 inhabitants [49] and there was an expansion of buildings even in extra-urban areas. The “Favara di Maredolce” complex, east of the city, was built. This involved the transformation of a marshy area into a fishpond extending 20 ha, with a 3.4 ha island [55] fed by the “Maredolce” and “San Ciro” springs. The network of canals that branched off the artificial basin allowed the cultivation of date palms and other fruit trees, especially citrus fruits, in the surrounding gardens [56]. Under Norman rule, the city grew further. Industry progressed, and trade increased. The population settled between 250,000 and 300,000 [49]. The Arab agricultural and cultural heritage was acquired by the Normans, who cultivated vegetable gardens and orchards in mixed cultivation in association with vines, which were well adapted to humid soils. Olives and mulberries were grown where the soil was drier. Non-irrigated agriculture (cereals and orchards with carob, olive, almond, and walnut trees) was practiced in the lands furthest from the city, toward the mountains [57].

Since the 13th century, the territories of “San Lorenzo”, “Ucciardone”, “Mezzomonreale”, and “Ficarazzi” were fiefdoms owned by noble families and ecclesiastical institutions, characterized as large estates leased without permanent plantations and only partially utilized as vegetable gardens [13]. Between 1360 and 1460, sugarcane was cultivated near the mouth of the Oreto River and along the course of the streams that descended from the mountains to the Cala. The entire Conca d’Oro was cultivated with irrigated vegetable gardens, non-irrigated crops (olive, carob, and almond trees), and vineyards. The plains of the hills were mainly cultivated with vineyards.

In the first half of the 13th century, interest in sugarcane cultivation was revived, which had an important economic significance in the 14th century [58]. During the 14th century, the landscape and cultivation techniques underwent few changes, and irrigated and intensive agriculture mainly concerned the territory between the Oreto River and the current “Porta di Termini”, where vegetable gardens were concentrated. The orchards remained substantially mixed [54]. Between the 13th and 15th centuries, administrative disorders and military events led to the economic decline of the city, which was reduced to no more than 40,000 inhabitants [49]. At the beginning of the 15th century, the city outside the walls was surrounded by many vineyards, vegetable gardens, mixed orchards, and, later, olive groves. The peri-urban landscape was characterized by beams, towers, and fortified rural houses to defend rural populations, springs, and crops [26]. During the 14th century, there was further development of fruit cultivation with the spread of new fruit varieties, such as apple, pear, peach, plum, fig, and apricot. Many flooded areas were left uncultivated to collect giant reeds (*Arundo donax*), which served as a support for vines and vegetable gardens and to build penthouses and separating walls of houses [13].

In the 15th century, citrus cultivation made important progress. A document dated 1413–1414 reports the first specialized citrus grove within the city in the Porta Carini district, where oranges, lemons, and lumia were grown [26]. Sugarcane cultivation took on an industrial connotation starting in 1420 to the detriment of vineyard areas. Plantations developed mainly towards the southeast, involving the lands of “La Cuba” toward Monreale and in the Santo Spirito Plain. In 1560, approximately 20 ha were cultivated in Palermo [58]. Sugarcane represented a revolution in the Palermo Plain because there was a transition from dry cultivation of wheat, barley, vines, and olives for the internal market to irrigated cultivation intended for export. The sugarcane industry had a period of maximum activity between 1541 and 1620 but ceased in 1690, mainly due to competition from sugar coming from America [54].

The end of the civil wars and the political stability established under Spanish rule led to a new increase in population. In 1591, the city had over 110,000 inhabitants [49]. At the beginning of the 16th century, the walled town was encircled by a mix of general and specialized orchards, a landscape that would persist throughout the century. Maps of the city [59,60,61] depict an abundance of wooded areas, specialized arboreta, and newly established private gardens. In keeping with tradition, these gardens served both productive and ornamental purposes [62]. With the disappearance of sugarcane cultivation, the land was converted into vineyards and, above all, olive groves [63]. Olive oil satisfied the need for fats that were no longer provided by pigs that were raised in the wild in the woods of the mountains surrounding the Conca that had been cut down in the previous century to obtain firewood for the extraction of cane sugar [64]. In the 17th century, there was a new demographic decline due to poor administration [49]. In the 17th century, the prickly pear spread first as an ornamental plant and then as a widespread crop in the Conca [26].

From 1712 to 1860, during the Bourbon government, Palermo enjoyed periods of true prosperity, which led to an increase in population [49]. In the 18th century, there was a noticeable increase in cultivation in the area closest to the city, marked by the appearance of new vegetable gardens and fields bordered by tree rows and orchards and enclosed by walls, known as “firríati” [43]. The Piana dei Colli and the area below Monreale were primarily covered with orchards and vineyards, although several uncultivated patches remained scattered among the cultivated lands [59,65]. At the end of the century, green areas arose for ornamental purposes. In 1777, Villa Giulia, the city’s first public garden, was inaugurated in the Piano di Sant’Erasmo, which was previously used for public executions. In 1789, the Botanical Garden was built next to Villa Giulia in an area occupied by crops and vineyards [66]. In 1799, the *Parco della Favorita*, the Bourbon Hunting Reserve, was established in an area occupied by woody fruit crops [46,57]. Many villas of the nobility in Palermo and Bagheria, completely new or originating from previous agricultural constructions, were equipped with ornamental green areas to attract visitors [67].

From the 18th century, the city’s expansion to the north was driven by noble families who built new villas and converted existing agricultural houses into summer residences [67]. This led to the creation of new hamlets to house the workers they employed. In the 19th century, with the Industrial Revolution, there was demographic growth. The development of trade favored the growth of the bourgeoisie, which further pushed the construction of villas and industrial areas and moved intensive cultivations for export outside the city limits.

In the early 20th century, the city had more than 330,000 inhabitants [49]. Further northern expansion into the fishing village of Mondello was driven by the wealthy bourgeoisie. This northern area, once used for orchards and the cultivation of citrus and vines, saw the development of the Valdesi-Mondello village in a large area that had been reclaimed from marshland [59]. The 1951 census, after the Second World War, records more than 500,000 inhabitants [68]. Currently, the city of Palermo has almost 650,000 inhabitants, slightly decreasing after the demographic peak of over 700,000 inhabitants in 1981 [68]. This trend is the natural consequence of the current period of economic/productive crisis [69].

### 3.2. Spatial Analysis

**Figure 2:**
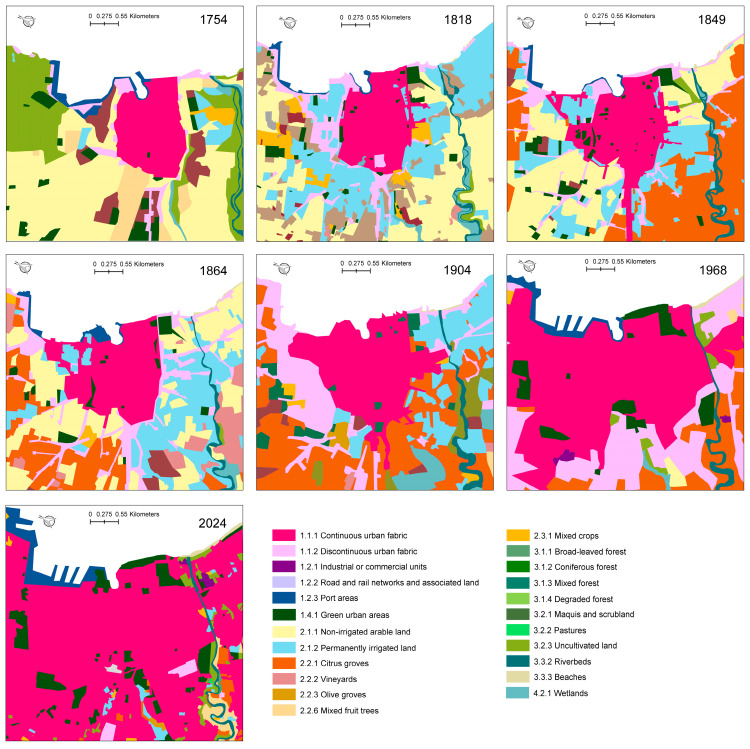
Analysis of land use in an area of about 17 km^2^ centered on the town of Palermo from 1754 to today. Classes according to Corine Land Cover [34].

The analysis of land use in an area of about 17 km^2^, centered on the town of Palermo (Figure 2, Table 2), showed that forests and semi-natural areas in 1754 covered 22.33%, and by 1818, the surface had decreased to below 3%. In the 20th century, it increased slightly to 6.35% and then settled at 4.35% today. Wetlands had a very limited surface area that reached a maximum of 3.78% in 1904. Agricultural areas increased starting in the 18th century from 49.66 to 67.40% in 1849 and then decreased until 1904 and collapsed in 1968. Artificial areas had the opposite trend, with values between 26.81% in 1754 and 44.93% in 1904, but then soared to 83.65% in 1968. The most substantial growth occurred in the 40 years following World War II, during which Palermo’s built-up area expanded dramatically from 600 to 7000 ha [70].

**Figure 3:**
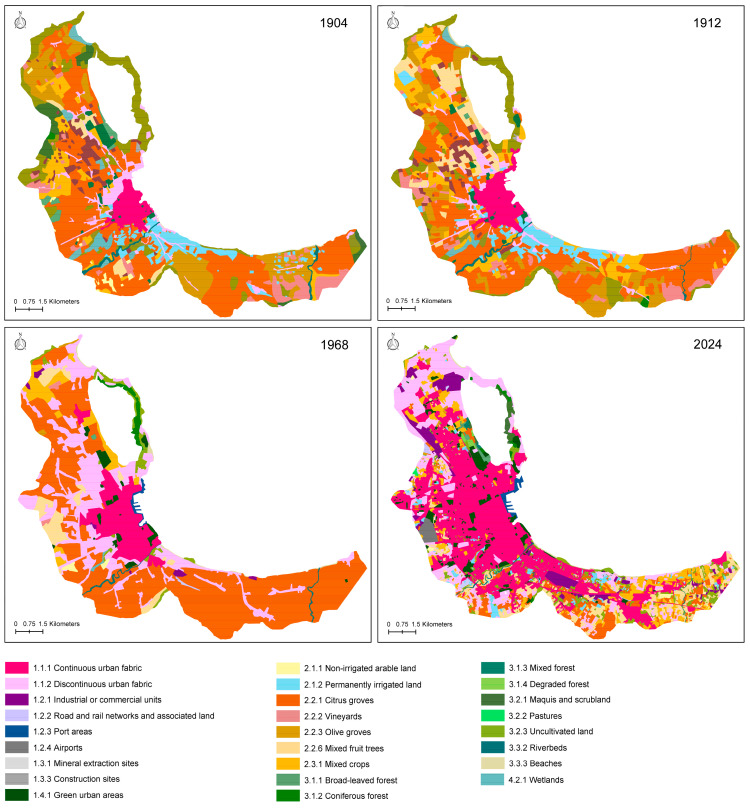
Analysis of land use throughout the whole Conca d’Oro from 1904 to today. Classes according to Corine Land Cover [34].

Among the punctual elements, the rectification of the terminal stretch of the Oreto River and the consequent loss of wetlands took place in the 19th century [71]. The study of the entire Conca d’Oro (Figure 3, Table 3) showed the same trend. In fact, forests and semi-natural areas were reduced from 17.7% in 1904 to 7.67% today, with a negative Annual Rate of Change until 1968 and a positive from 1968 to 2024 (Table 4). Wetlands decreased from 36.55% in 1904 to 15.05% in 2024. For this class, too, there was a positive trend between 1968 and 2024 (Table 4). Agricultural areas increased from 69.16% in 1904 to 75.45% in 1912 and then decreased to 26.01% in 2024. Artificial surfaces remained below 12% until 1912, increasing to 35.52% in 1868 and to 66% in 2024.

Overall, since 1750, there has been a progressive transformation of semi-natural areas into agricultural areas and later into artificial areas. The inhabited center of Palermo remained confined within the perimeter walls until the early 1900s and underwent a rapid expansion after World War II. In 1754, arable land and tree crops prevailed (we have no data relating to irrigated surfaces). The 1818 map shows vegetable gardens all around the inhabited center. In the 1849 map, many arable lands to the south of the inhabited center were converted into citrus groves. The map drawn in 1864 showed new citrus groves in the northwest of the city. Between 1864 and 1904, the city of Palermo expanded along the roads leading toward the countryside, initially as low-density urban factories and then as continuous high-density urban factories. In 1904, a map reported the prevalence of vegetable gardens along the coast from the Oreto River toward the east. The area between Palermo and Bagheria was mainly occupied by citrus groves. In 1968, the map highlights the uniform expansion of the low-density urban fabric around the initial nucleus and the dominance of citrus groves among agricultural areas. The current map shows a prevalence of a high-density urban fabric; the citrus groves have remained almost exclusively on the slopes of the mountains southeast of Palermo, replaced elsewhere by low-density urban factories.

The analysis of Ecosystem Service Values (Table 5, Table 6 and Table 7) highlights an overall loss of EUR 237.57 million from 1864 to today, with an overall negative trend from 1864 to 1968 and an improvement from 1968 to today. This general trend is due to the increase in artificial surfaces, which have a low ecosystem value, and to the decrease in classes with higher values (agricultural areas, semi-natural areas, but especially wetlands).

### 3.3. Floristic Study

**Figure 4:**
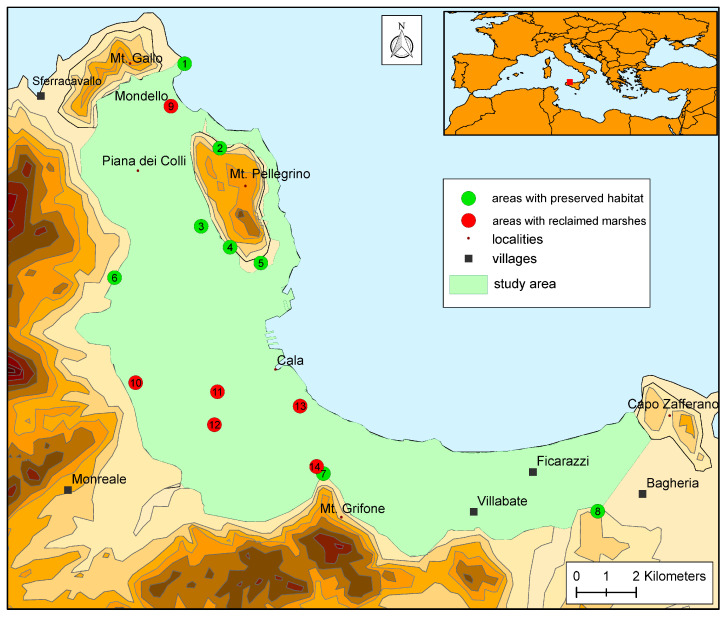
Areas with preserved habitat (in green) and areas with reclaimed marshes (in red) in the investigated area: (1) see rock in Piano Gallo; (2) north slope cliffs of Mt. Pellegrino; (3) Bosco Niscemi; (4) west slope cliffs of Mt. Pellegrino; (5) south slope cliffs of Mt. Pellegrino; (6) cliffs of Baida; (7) cliffs of Mt. Grifone; (8) cliffs of Mt. Giancaldo; (9) marshes of Mondello; (10) marshes of Boccadifalco; (11) marshes of Danisinni; (12) marshes of Porrazzi; (13) marshes at the mouth of Oreto River; (14) marshes of San Ciro.

In the Conca d’Oro, 18 endemic taxa have been found, 8 of which are endemic to Italy and 10 to Sicily (Table 8). Nine species grow in rocky habitats and are scattered on the cliffs of the mountains that surround the Conca d’Oro, but their occurrence in the plain is only marginal (Figure 4). Eight taxa are related to pastures and uncultivated lands. *Limonium bocconei* (Lojac.) Litard. grows on sea rocks. *Antirrhinum siculum* Mill. and *Biscutella maritima* Ten. are scattered throughout the whole area. *Euphorbia ceratocarpa* Ten. occurs near Baida and the Eleuterio River. The Oreto River is the locus classicus of *Carex panormitana* Guss., a species occurring in Sicily, Sardinia, and Tunisia [72]. It also hosts a population of *Platanus orientalis* L. at the extreme western limit of this species’ range [73].

In total, 40 taxa historically reported for the Conca d’Oro are no longer present in the territory (Table 9).

These are mainly related to humid environments, beaches, and, to a lesser extent, to cultivated and uncultivated dry land. The areas that have undergone the greatest transformations due to reclamation, which have led to habitat loss, are the Mondello marshes, the marshes at the mouth of the Oreto River, and the wetlands of San Ciro and Baida. In some cases (e.g., *Descurainia sophia*, *Elatine macropoda*, *Stuckenia pectinata*), the local extinction is due to the change in agronomic techniques, which has determined the disappearance of ditches where the species grew. Many of the disappeared species have been reported for these areas (Figure 4). Another floristic contingent that has disappeared from the area is that linked to sandy coasts, such as *Cressa cretica* L., which has been assessed as endangered in Italy [80], *Echium arenarium* Guss., and *Sporobolus pungens* (Schreb.) Kunth. *Descurainia sophia* (L.) Webb ex Prantl, *Elatine macropoda* Guss., and *Stuckenia pectinata* (L.) Börner are species related to watered grounds and irrigation canals that disappeared from the Conca d’Oro when irrigated, cultivated fields became more rare.

## 4. Discussion

The following upheavals have marked the plant landscape of the Conca d’Oro of Palermo: the deforestation of the holm oak forests for the cultivation of cereals that occurred during Roman domination (254 BC–444); the reduction in cereal crops and uncultivated areas following the advent of irrigated agriculture in the 9th century with the Arabs; the disappearance of humid areas following the reclamation that took place in the 16th century; the outbreak of citrus monoculture (lemon and mandarin) that occurred in the first half of the 19th century [81]; and the loss of agricultural areas following building expansion after World War II. As already underlined for other areas in Europe [82], the changes in inhabited centers and their surroundings are the direct result of specific political and economic decisions. These policies produce land use changes that may lead to unexpected consequences, including biodiversity loss and soil degradation [83]. Adopting a historical perspective on land and water use can be valuable for reassessing the reasons behind policy creation, understanding their unintended consequences, and identifying potential improvements [84].

The location of different crops is strongly influenced by soil characteristics and water availability [85]. The almost total disappearance of humid and dune habitats is a consequence of the increase in land use. Pasammophilous, halohygrophilous, and hydrophytic habitats in Italy are considered the most threatened by extinction [86]. In the Conca d’Oro, the humid habitats were modified and destroyed first, then the beaches, and finally, dry meadows and traditional extensive agricultural areas. Some rocky areas and cliffs surrounding the Conca d’Oro (Figure 4, areas 1–8) are true reserves of biodiversity for species adapted to this habitat. It is not possible to restore the destroyed wetlands (Figure 4, areas 9–13) because now the city rises where there were the marshes. Only for the marshes of San Ciro (Figure 4, area 14), at the edge of the town, can habitat restoration be foreseen when restoring the Maredolce castle with its adjoining lake.

The Oreto River remains an important reservoir of plant diversity. Due to its canyons in carbonate rock, in some places, it has preserved more natural stretches that still host plant communities of great biogeographic interest. In contrast, with its gentle morphologies dug into the clay and its banks, the Eleuterio River is cultivated up to the riverbed. The least altered areas are those that remain uncultivated.

Balancing development and ecological protection in urbanization requires a multidisciplinary approach that integrates sustainable planning, green economics, ecosystem services, and robust governance [87]. By promoting sustainable urban growth, investing in environmental conservation, and fostering community participation, cities can thrive while protecting their natural heritage. The growth of the city of Palermo has had a strong impact on the surrounding environment. Sustainable urban planning focuses on growth that minimizes ecological impacts. As already observed for other Italian coastal cities [88], the ecosystem services provision is moving from the inner urban core toward the peri-urban and rural areas. The integration of green spaces such as parks and green roofs can help promote biodiversity, improving both the quality of life and environmental conditions in urban spaces [89]. Urban planners should integrate ecosystem services into urban development strategies by maintaining wetlands or increasing the number and surface of green areas [90]. Currently, the Conca d’Oro has a percentage of green urban areas of 4.50%, more than double compared to 1968. Palermo has a notably low density of urban green spaces in comparison to other Italian cities with populations exceeding 200,000 [91]. The current master plan for the city of Palermo [92] does not provide for further urban development. Only urban redevelopment is allowed. This activity is subject to linear and aerial constraints that protect sites of community importance, archaeological assets, woods, the coastal strip, etc.

## 5. Conclusions

This survey examined the landscape dynamics and the natural plant heritage of the Conca d’Oro of Palermo from the first settlements to the present, highlighting spatial changes driven by both local and distant factors. An analysis of historical documents and maps revealed that changes in the plant landscape were closely linked to political and economic developments. Despite inherent limitations and necessary assumptions, historical maps provide valuable insights into long-term spatial trajectories and land cover changes. This research underscores the significance and constraints of historical sources in understanding landscape change processes in urban areas. Additionally, it emphasizes the need to conserve and valorize micro-areas of high biodiversity, such as cliffs and river canyons, which serve as biodiversity reservoirs and offer direct contact with nature to city inhabitants. From a future perspective, there is the need for (1) long-term ecological monitoring of the Conca d’Oro area, also in the context of climate change, (2) ongoing tracking of shifts in the distribution of plant species of conservation concern, (3) evaluation of the relationship between urban expansion and biodiversity decline, and (4) more refined trend projections that integrate current technologies and models.

## Figures and Tables

**Table 1:** Main events in the history of the city of Palermo (from [43,44]).

Dates	Event
8th century BC	First urban nucleus
7th century BC–254 BC	Carthaginians settlement
277 BC	One year of Greek settlement
254 BC–444	Romans
444–535	Vandals
535–830	Byzantines
830–1071	Arabs
1071–1266	Normans
1266–1882	Angevins
1282–1412	Aragonese
1412–1717	Spanish viceroys
1717–1734	Savoy
1734–1861	Bourbons
1861	Annexation to Italy
1943	World War II bombings
1952–1962	Urban sprawl (Sack of Palermo)

**Table 2:** Analysis of land use in an area of about 17 km^2^ centered on the town of Palermo from 1754 until today. Classes are reported according to Corine Land Cover [34].

**Land Uses**	**2024**		**1968**		**1904**		**1864**	
	**Km^2^**	**%**	**Km^2^**	**%**	**Km^2^**	**%**	**Km^2^**	**%**
1.1.1 Continuous urban fabric	13.28	76.63	8.89	52.12	3.88	23.75	4.43	17.19
1.1.2 Discontinuous urban fabric	0.20	1.17	3.91	22.93	2.88	17.65	2.89	11.21
1.2.1 Industrial or commercial units	0.06	0.37	0.09	0.53	0.00	0.00	0.00	0.00
1.2.2 Road, rail networks, and associated land	0.00	0.00	0.00	0.00	0.00	0.00	0.00	0.00
1.2.3 Port areas	0.64	3.69	0.43	2.50	0.00	0.00	0.31	1.19
1.2.4 Airports	0.00	0.00	0.00	0.00	0.00	0.00	0.00	0.00
1.3.1 Mineral extraction sites	0.04	0.22	0.00	0.00	0.00	0.00	0.00	0.00
1.3.3 Construction sites	0.00	0.00	0.00	0.00	0.00	0.00	0.00	0.00
1.4.1 Green urban areas	1.37	7.92	0.95	5.58	0.58	3.53	0.60	2.31
**Artificial surfaces**	**15.60**	**89.99**	**14.27**	**83.65**	**7.34**	**44.93**	**8.22**	**31.89**
2.1.1 Non-irrigated arable land	0.00	0.00	0.13	0.79	0.00	0.00	6.37	24.72
2.1.2 Permanently irrigated land	0.28	1.61	0.00	0.00	2.14	13.07	4.65	18.05
2.2.1 Citrus groves	0.28	1.63	1.83	10.74	4.72	28.90	3.78	14.68
2.2.2 Vineyards	0.00	0.00	0.00	0.00	0.09	0.57	1.96	7.58
2.2.3 Olive groves	0.04	0.22	0.00	0.00	0.15	0.92	0.00	0.00
2.2.6 Mixed orchard	0.30	1.72	0.05	0.32	0.08	0.50	0.00	0.00
2.3.1 Complex agricultural systems	0.08	0.47	0.05	0.29	0.16	0.96	0.19	0.74
**Agricultural areas**	**0.98**	**5.65**	**2.07**	**12.13**	**7.34**	**44.93**	**16.96**	**65.77**
3.1.1 Broad-leaved forest	0.02	0.13	0.00	0.00	0.00	0.00	0.00	0.00
3.1.2 Coniferous forest	0.00	0.00	0.00	0.00	0.00	0.00	0.00	0.00
3.1.3 Mixed forest	0.00	0.00	0.00	0.00	0.00	0.00	0.00	0.00
3.1.4 Degraded forest	0.00	0.00	0.00	0.00	0.00	0.00	0.00	0.00
3.2.1 Maquis	0.00	0.00	0.00	0.00	0.00	0.00	0.00	0.00
3.2.2 Pasture	0.01	0.04	0.00	0.00	0.00	0.00	0.00	0.00
3.2.3 Uncultivated land	0.42	2.45	0.30	1.75	0.61	3.75	0.05	0.18
3.3.2 Riverbeds	0.21	1.22	0.29	1.72	0.43	2.61	0.34	1.32
3.3.3 Beaches	0.09	0.51	0.04	0.25	0.00	0.00	0.04	0.17
**Forest and semi-natural areas**	**0.75**	**4.35**	**0.63**	**3.72**	**1.04**	**6.36**	**0.43**	**1.67**
4.2.1 Marshes	0.00	0.00	0.09	0.51	0.62	3.78	0.17	0.66
**Wetlands**	**0.00**	**0.00**	**0.09**	**0.51**	**0.62**	**3.78**	**0.17**	**0.66**
**TOTAL**	**17.33**		**17.07**		**16.33**		**25.78**	
**Land Uses**	**1849**		**1818**		**1754**			
	**Km^2^**	**%**	**Km^2^**	**%**	**Km^2^**		**%**	
1.1.1 Continuous urban fabric	2.77	17.16	2.40	16.94	2.08		13.92	
1.1.2 Discontinuous urban fabric	1.31	8.11	1.15	8.10	0.82		5.52	
1.2.1 Industrial or commercial units	0.00	0.00	0.00	0.00	0.00		0.00	
1.2.2 Road, rail networks, and associated land	0.00	0.00	0.00	0.00	0.00		0.00	
1.2.3 Port areas	0.11	0.69	0.12	0.83	0.41		2.77	
1.2.4 Airports	0.00	0.00	0.00	0.00	0.00		0.00	
1.3.1 Mineral extraction sites	0.00	0.00	0.00	0.00	0.00		0.00	
1.3.3 Construction sites	0.00	0.00	0.05	0.35	0.00		0.00	
1.4.1 Green urban areas	0.57	3.54	0.45	3.19	0.69		4.60	
**Artificial surfaces**	**4.76**	**29.50**	**4.17**	**29.40**	**4.00**		**26.81**	
2.1.1 Non-irrigated arable land	3.94	24.39	4.86	34.26	6.05		40.49	
2.1.2 Permanently irrigated land	2.49	15.41	3.79	26.74	0.24		1.58	
2.2.1 Citrus groves	4.46	27.61	0.00	0.00	0.00		0.00	
2.2.2 Vineyards	0.00	0.00	0.08	0.55	0.00		0.00	
2.2.3 Olive groves	0.00	0.00	0.00	0.00	0.00		0.00	
2.2.6 Mixed orchard	0.00	0.00	0.00	0.00	0.96		6.42	
2.3.1 Complex agricultural systems	0.00	0.00	0.61	4.33	0.18		1.18	
**Agricultural areas**	**10.88**	**67.40**	**9.34**	**65.88**	**7.42**		**49.66**	
3.1.1 Broad-leaved forest	0.00	0.00	0.00	0.00	0.00		0.00	
3.1.2 Coniferous forest	0.00	0.00	0.00	0.00	0.00		0.00	
3.1.3 Mixed forest	0.00	0.00	0.00	0.00	0.00		0.00	
3.1.4 Degraded forest	0.00	0.00	0.00	0.00	0.00		0.00	
3.2.1 Maquis	0.00	0.00	0.00	0.00	0.00		0.00	
3.2.2 Pasture	0.00	0.00	0.00	0.00	0.00		0.00	
3.2.3 Uncultivated land	0.09	0.59	0.10	0.73	3.13		20.94	
3.3.2 Riverbeds	0.36	2.22	0.23	1.62	0.21		1.38	
3.3.3 Beaches	0.02	0.15	0.01	0.07	0.00		0.00	
**Forest and semi-natural areas**	**0.48**	**2.95**	**0.34**	**2.42**	**3.33**		**22.33**	
4.2.1 Marshes	0.02	0.14	0.32	2.29	0.18		1.21	
**Wetlands**	**0.02**	**0.14**	**0.32**	**2.29**	**0.18**		**1.21**	
**TOTAL**	**16.14**		**14.18**		**14.94**			

**Table 3:** Analysis of land use throughout the whole Conca d’Oro from 1864 until today. Classes are reported according to Corine Land Cover [34].

Land Uses	2024		1968		1904		1864	
	Km^2^	%	Km^2^	%	Km^2^	%	Km^2^	%
1.1.1 Continuous urban fabric	38.44	37.72	10.52	10.38	5.50	5.62	3.88	3.97
1.1.2 Discontinuous urban fabric	17.71	17.37	22.48	22.18	4.90	5.01	5.47	5.60
1.2.1 Industrial or commercial units	4.80	4.71	0.60	0.60	0.00	0.00	0.00	0.00
1.2.2 Road, rail networks, and associated land	0.55	0.54	0.14	0.14	0.00	0.00	0.00	0.00
1.2.3 Port areas	0.64	0.63	0.43	0.42	0.00	0.00	0.00	0.00
1.2.4 Airports	0.94	0.92	0.00	0.00	0.00	0.00	0.00	0.00
1.3.1 Mineral extraction sites	0.05	0.05	0.00	0.00	0.00	0.00	0.00	0.00
1.3.3 Construction sites	0.02	0.02	0.00	0.00	0.06	0.06	0.11	0.11
1.4.1 Green urban areas	4.58	4.50	1.83	1.80	1.23	1.26	1.74	1.78
**Artificial surfaces**	**67.74**	**66.46**	**36.01**	**35.52**	**11.68**	**11.95**	**11.20**	**11.46**
2.1.1 Non-irrigated arable land	0.39	0.38	0.39	0.38	0.00	0.00	0.75	0.77
2.1.2 Permanently irrigated land	2.37	2.33	0.00	0.00	5.80	5.93	5.53	5.66
2.2.1 Citrus groves	7.72	7.57	51.86	51.15	35.97	36.80	36.15	36.99
2.2.2 Vineyards	0.04	0.04	0.31	0.30	4.58	4.69	5.55	5.67
2.2.3 Olive groves	0.45	0.44	0.22	0.21	10.97	11.22	12.31	12.59
2.2.6 Mixed orchard	7.25	7.12	4.05	3.99	6.15	6.30	1.56	1.60
2.3.1 Complex agricultural systems	8.29	8.13	3.93	3.87	10.27	10.51	5.75	5.88
**Agricultural areas**	**26.52**	**26.01**	**60.75**	**59.92**	**73.74**	**75.45**	**67.60**	**69.16**
3.1.1 Broad-leaved forest	0.42	0.41	0.07	0.07	0.14	0.15	1.02	1.04
3.1.2 Coniferous forest	0.43	0.43	1.09	1.08	0.20	0.20	0.14	0.14
3.1.3 Mixed forest	0.17	0.17	0.00	0.00	0.00	0.00	0.00	0.00
3.1.4 Degraded forest	0.25	0.24	0.00	0.00	0.00	0.00	0.00	0.00
3.2.1 Maquis	0.84	0.82	0.00	0.00	0.00	0.00	2.85	2.91
3.2.2 Pasture	0.09	0.09	0.00	0.00	0.00	0.00	0.00	0.00
3.2.3 Uncultivated land	4.69	4.60	2.28	2.25	9.93	10.16	11.75	12.02
3.3.2 Riverbeds	0.39	0.38	0.64	0.63	0.80	0.82	1.03	1.06
3.3.3 Beaches	0.40	0.39	0.39	0.39	0.25	0.26	0.00	0.00
**Forest and semi-natural areas**	**7.67**	**7.53**	**4.47**	**4.41**	**11.32**	**11.59**	**16.78**	**17.17**
4.2.1 Marshes	0.00	0.00	0.16	0.16	0.99	1.01	2.16	2.21
**Wetlands**	**15.34**	**15.05**	**9.10**	**8.97**	**23.64**	**24.19**	**35.73**	**36.55**
**TOTAL**	**101.93**		**101.39**		**97.74**		**97.74**	

**Table 4:** Land cover change in different periods and annual rate of change throughout the whole Conca d’Oro from 1864 until today. Classes follow Table 3.

Classes of Land Uses	Change (1864–1904) % ^a^	Change (1904–1968) % ^a^	Change (1968–2024) % ^a^	Annual Rate of Change (1864–1904) % ^b^	Annual Rate of Change (1904–1968) % ^b^	Annual Rate of Change (1968–2024) % ^b^
Artificial surfaces	0.49	23.57	30.94	0.11	3.25	1.57
Agricultural areas	6.29	−15.53	−33.91	0.23	−0.28	−1.01
Forest and semi-natural areas	−5.58	−7.18	3.12	−0.81	−0.95	1.28
Wetlands	−12.36	−15.22	6.08	−0.85	−0.96	1.22

^a^ Percentage change in the component; ^b^ Percentage of the annual rate of change in each class.

**Table 5:** Land cover area (in hectares), Ecosystem Service Values (in EUR per hectare from [38]), value (in millions of EUR) for years throughout the whole Conca d’Oro from 1864 until today. Classes follow Table 3.

Land Uses	2024 (ha)	1968 (ha)	1904 (ha)	1864 (ha)	Value (EUR/ha)	2024 (M EUR)	1968 (M EUR)	1904 (M EUR)	1864 (M EUR)
1.1.1 Continuous urban fabric	3844	1052	550	388	90.1	0.35	0.09	0.05	0.03
1.1.2 Discontinuous urban fabric	1771	2248	490	547	270.3	0.48	0.61	0.13	0.15
1.2.1 Industrial or commercial units	480	60	0	0	90.1	0.04	0.01	0.00	0.00
1.2.2 Road, rail networks, and associated land	55	14	0	0	90.1	0.00	0.00	0.00	0.00
1.2.3 Port areas	64	43	0	0	90.1	0.01	0.00	0.00	0.00
1.2.4 Airports	94	0	0	0	90.1	0.01	0.00	0.00	0.00
1.3.1 Mineral extraction sites	5	0	0	0	90.1	0.00	0.00	0.00	0.00
1.3.3 Construction sites	2	0	6	11	90.1	0.00	0.00	0.00	0.00
1.4.1 Green urban areas	458	183	123	174	270.3	0.12	0.05	0.03	0.05
**Artificial surfaces**	**6774**	**3601**	**1168**	**1120**		**1.01**	**0.76**	**0.22**	**0.23**
2.1.1 Non-irrigated arable land	39	39	0	75	520.4	0.02	0.02	0.00	0.04
2.1.2 Permanently irrigated land	237	0	580	553	260.2	0.06	0.00	0.15	0.14
2.2.1 Citrus groves	772	5186	3597	3615	260.2	0.20	1.35	0.94	0.94
2.2.2 Vineyards	4	31	458	555	260.2	0.00	0.01	0.12	0.14
2.2.3 Olive groves	45	22	1097	1231	260.2	0.01	0.01	0.29	0.32
2.2.6 Mixed orchard	725	405	615	156	260.2	0.19	0.11	0.16	0.04
2.3.1 Complex agricultural systems	829	393	1027	575	260.2	0.22	0.10	0.27	0.15
**Agricultural areas**	**2652**	**6075**	**7374**	**676**		**0.70**	**1.59**	**1.92**	**1.78**
3.1.1 Broad-leaved forest	42	7	14	102	803.6	0.03	0.01	0.01	0.08
3.1.2 Coniferous forest	43	109	20	14	803.6	0.03	0.09	0.02	0.01
3.1.3 Mixed forest	17	0	0	0	803.6	0.01	0.00	0.00	0.00
3.1.4 Degraded forest	25	0	0	0	810.7	0.02	0.00	0.00	0.00
3.2.1 Maquis	84	0	0	285	810.7	0.07	0.00	0.00	0.23
3.2.2 Pasture	9	0	0	0	550.4	0.00	0.00	0.00	0.00
3.2.3 Uncultivated land	469	228	993	1175	550.4	0.26	0.13	0.55	0.65
3.3.2 Riverbeds	39	64	80	103	830.7	0.03	0.05	0.07	0.09
3.3.3 Beaches	40	39	25	0	740.6	0.03	0.03	0.02	0.00
**Forest and semi-natural areas**	**767**	**447**	**1132**	**1678**		**0.50**	**0.30**	**0.66**	**1.06**
4.2.1 Marshes	0	16	99	216	116,091	0.00	1.86	11.49	25.08
**Wetlands**	**1534**	**910**	**2364**	**3573**	**116,091**	**178.08**	**105.64**	**274.44**	**414.79**
**TOTAL**	**10,193**	**10,139**	**9774**	**9774**		**180.29**	**108.30**	**277.23**	**417.86**

**Table 6:** Ecosystem Services Values changes (millions of EUR) throughout the whole Conca d’Oro from 1864 until today. Classes follow Table 3.

Classes of Land Uses	Change (1864–1904) (M EUR)	Change (1904–1968) (M EUR)	Change (1968–2024) (M EUR)	Change (1864–2024) (M EUR)
Artificial surfaces	−0.02	0.55	0.25	0.78
Agricultural areas	0.14	−0.33	−0.89	−1.08
Forest and semi-natural areas	−0.40	−0.36	0.19	−0.56
Wetlands	−140.35	−168.80	72.44	−236.71
TOTAL	−140.63	−168.94	71.99	−237.57

**Table 7:** Percentage variations in Ecosystem Services Values throughout the whole Conca d’Oro from 1864 until today. Classes follow Table 3.

Classes of Land Uses	Change (1864–1904) % ^a^	Change (1904–1968) % ^a^	Change (1968–2024) % ^a^	Change (1864–2024) % ^a^
Artificial surfaces	−6.52	253.32	32.72	338.37
Agricultural areas	7.89	−17.07	−56.01	−60.64
Forest and semi-natural areas	−37.64	−54.35	64.73	−53.11
Wetlands	−33.84	−61.51	68.57	−57.07

^a^ Percentage change in the component.

**Table 8:** Chorothype, localities, and habitats of endemic taxa occurring in the investigated area.

Taxon	Chorotype	Localities	Habitat
*Antirrhinum siculum* Mill.	Endem. Ital.	the whole area	rocks, walls
*Biscutella maritima* Ten.	Endem. Ital.	the whole area	fields, uncultivated lands
*Brassica rupestris* Raf. subsp. *rupestris*	Endem. Ital.	bases of Mt. Pellegrino, Mt. Gallo, Mt. Grifone, Bagheria	rocks
*Carlina sicula* Ten. subsp. *sicula*	Endem. Sic.	bases of Mt. Pellegrino, Mt. Gallo, and Mt. Grifone	dry pastures
*Centaurea panormitana* Lojac.	Endem. Sic.	bases of Mt. Pellegrino, Mt. Gallo, and Mt. Grifone	rocks
*Centaurea todaroi* Lacaita	Endem. Sic.	Bagheria	rocks
*Cymbalaria pubescens* (C.Presl) Cufod.	Endem. Sic.	base of Mt. Pellegrino	rocks
*Euphorbia ceratocarpa* Ten.	Endem. Sic.	Baida, Eleuterio River	uncultivated lands
*Hexaphylla rupestris* (Tineo) P.Caputo and Del Guacchio	Endem. Sic.	base of Mt. Pellegrino	rocks
*Lathyrus odoratus* L.	Endem. Ital.	Baida	uncultivated lands
*Limonium bocconei* (Lojac.) Litard.	Endem. Sic.	Mondello	sea rocks
*Micromeria graeca* subsp. *fruticulosa* (Bertol.) Guinea	Endem. Ital.	bases of Mt. Pellegrino, Mt. Gallo, and Mt. Grifone	rocks
*Micromeria graeca* subsp. *tenuifolia* (Ten.) Nyman	Endem. Ital.	bases of Mt. Pellegrino, Mt. Gallo, and Mt. Grifone	rocks
*Ophrys sphegodes* subsp. *panormitana* (Tod.) E.Nelson	Endem. Ital.	Boccadifalco	rocky pastures
*Orchis brancifortii* Biv.	Endem. Ital.	bases of Mt. Pellegrino, Mt. Gallo, and Mt. Grifone	rocky pastures
*Pimpinella gussonei* (C.Presl) Bertol.	Endem. Sic.	bases of Mt. Pellegrino, Mt. Gallo	rocky pastures
*Romulea linaresii* Parl. subsp. *linaresii*	Endem. Sic.	base of Mt. Pellegrino	rocky pastures
*Seseli bocconei* Guss.	Endem. Sic.	base of Mt. Pellegrino	rocks

**Table 9:** Taxa no longer found in the territory of the Conca d’Oro, their chorotype, localities, habitat, the last data of record and references or voucher specimens kept in the *Herbarium Mediterraneum Panormitanum* (PAL).

Taxon	Chorotype	Localities	Habitat	Century of Last Record and References	Possible Causes of Extinction
*Alopecurus bulbosus* Gouan subsp. *bulbosus*	Eurimedit.	Mondello	Coastal marshes	19th [74,75]	Reclamation
*Althaea officinalis* L.	Subcosmop.	City center; Mondello	Ditches	19th [74,76]	Change of cultivation techniques
*Anchusa undulata* subsp. *hybrida* (Ten.) Bég.	Stenomedit.	Arenella and Vergine Maria	Uncultivated lands	19th [74]	Constructions
*Artemisia campestris* subsp. *variabilis* (Ten.) Greuter	Endem. Ital.	Mondello	Ditches	19th [76]	Change of cultivation techniques
*Bolboschoenus maritimus* (L.) Palla	Cosmop.	Baida, Mondello	Marshes	19th [75]	Reclamation
*Calamagrostis arenaria* subsp. *arundinacea* (Husn.) Banfi, Galasso & Bartolucci	Eurimedit.	Mondello	Sandy dunes	19th PAL74487; PAL16517	Constructions
*Callitriche brutia* Petagna	Subatlant.	Mondello	Seasonal wetlands	19th [74]	Reclamation
*Callitriche palustris* L.	Circumbor.	Mondello	Seasonal wetlands	19th [75]	Reclamation
*Callitriche truncata* Guss. subsp. *truncata*	Subatlant.	Arenella, Vergine Maria	Wetlands	19th [76]	Reclamation
*Catabrosa aquatica* (L.) P.Beauv.	Circumbor.	Roccazzo	Marshes	19th [75]	Reclamation
*Cressa cretica* L.	Subcosmop.	Sferracavallo	Beaches, brackish wetlands	19th [74]	Reclamation
*Cyperus capitatus* Vand.	Stenomedit.	Fondachelli, Mondello, Villabate	Sandy dunes	19th [75]	Constructions
*Cyperus fuscus* L.	Paleotemp.	San Ciro	Seasonally wet areas	19th [75]	Reclamation
*Cyperus papyrus* L.	African	Papireto river	Riverbanks	10th [31]	Constructions
*Descurainia sophia* (L.) Webb ex Prantl	Subcosmop.	Oreto river	Vegetable gardens	19th [74]	Change of cultivation techniques
*Echium arenarium* Guss.	Stenomedit.	Vergine Maria	Beaches	19th PAL 64807; [76]	Habitat alteration
*Elatine macropoda* Guss.	Stenomedit.	Mondello	Irrigation ducts	19th PAL 79149; [74]	Change of cultivation techniques
*Groenlandia densa* (L.) Fourr.	Eurosiber.	San Ciro	Water pools	19th [76]	Reclamation
*Humulus lupulus* L.	Paleotemp.	Porrazzi	Riverbanks	19th [74]	Constructions
*Hypericum pubescens* Boiss.	W-Stenomedit.	Mondello	Wetlands, springs	19th [76]	Reclamation
*Ipomoea sagittata* Poir.	Anfiatlant.	Mondello	Marshes	19th [76]	Reclamation
*Isolepis cernua* (Vahl) Roem. & Schult.	Subcosmop.	Mondello, San Ciro, Villabate, Roccazzo, Boccadifalco	Ponds, marshes	19th [75,76]	Reclamations
*Juncus acutiflorus* Ehrh. ex Hoffm.	Europ.	Baida, Roccazzo	Marshes	19th PAL 50041; [74]	Reclamation
*Myosotis sylvatica* subsp. *subarvensis* Grau	Europ.	Mondello	Wet soils	19th [76]	Reclamation
*Nanozostera noltei* (Hornem.) Toml. & Posl.	Subatlant., Medit.	Mondello	Coastal lagoons	19th [76]	Reclamation
*Potamogeton pusillus* L.	Subcosmop.	Oreto river	Lakes, rivers	19th [74]	Water pollution
*Rumex palustris* Sm.	Eurasiat.	Boccadifalco	Marshes	19th [76]	Reclamation
*Saccharum biflorum* Forssk.	Paleotrop.	Fiume Oreto at bridge Corleone and bridge della Grazia	Seasonally moist soils	19th [75]	Replaced by *Arundo donax* L.
*Salicornia fruticosa* (L.) L.	Eurimedit.	Arenella, Mondello	Salt marshes	19th [75]	Reclamation
*Salvia fruticosa* subsp. *thomasii* (Lacaita) Brullo, Guglielmo, Pavone & Terrasi	Endem. Ital.	Piana dei Colli	Arid grasslands	19th [75]	Constructions
*Salvia viridis* L.	Stenomedit.	Base of Mt Gallo	Arid grasslands	19th PAL79788	Constructions
*Schoenus nigricans* L.	Subcosmop.	Boccadifalco, Mondello	Wet meadows	19th PAL49888; [75]	Reclamation
*Scirpoides holoschoenus* (L.) Soják	Eurimedit.	Mondello, San Ciro	Wetlands	19th [75]	Reclamation
*Solenopsis bivonae* (Tineo) M.B.Crespo, Serra & Juan	Stenomedit.	Guadagna, Mondello, city center	Seasonally wet soil	19th [74,76]	Reclamation
*Solenopsis laurentia* (L.) C. Presl	Stenomedit.	Mondello	Seasonally wet soil	19th [77,78]	Reclamation
*Sporobolus pungens* (Schreb.) Kunth	Pantrop.	Romagnolo	Beaches	19th [75,79]	Habitat alteration
*Stuckenia pectinata* (L.) Börner	Subcosmop.	Mondello	Irrigation ducts	19th [75]	Change of cultivation techniques
*Bulliarda vaillantii* (Willd.) DC.	Subatlant.	Mondello	Seasonally wet soil	19th [74]	Reclamation
*Thinopyrum elongatum* (Host) D.R.Dewey	Eurimedit.	Mondello	Beaches	19th PAL74026	Habitat alteration
*Veronica anagalloides* Guss.	Euromedit.	Mondello	Marshes	19th PAL43759; [75]	Reclamations

For sources published over multiple years, only the first year of publication is included in the references.

## Data Availability

The original contributions presented in the study are included in the text; further inquiries can be directed to the corresponding author.
